# Efficacy of an injectable toltrazuril – gleptoferron (Forceris^®^) to control coccidiosis (*Cystoisospora suis*) in comparison with iron supplemented piglets without anticoccidial treatment

**DOI:** 10.1016/j.vpoa.2019.100002

**Published:** 2019-01-29

**Authors:** Lysanne Hiob, Ivette Holzhausen, Daniel Sperling, Gaëlle Pagny, Laurianne Meppiel, Naomi Isaka, Arwid Daugschies

**Affiliations:** a Albrecht - Daniel - Thaer - Institute for Agricultural Sciences e.V. at University of Leipzig, An den Tierkliniken 29, 04103 Leipzig, Germany; b Institute of Parasitology, Faculty of Veterinary Medicine, University of Leipzig, An den Tierkliniken 35, 04103 Leipzig, Germany; c CEVA Santé Animale, 10 avenue de la Ballastière, 33500 Libourne, France

**Keywords:** AUC, area under the curve, BW, body weight, CP, control group, FS, faecal score, MDBWG, mean daily body weight gain, OPG, oocysts per gram of faeces, SD, study day, Piglet, Coccidiosis, *Cystoisospora suis*, Toltrazuril, Forceris^®^, Natural infection, Diarrhoea

## Abstract

The efficacy and safety of an injectable combination product containing toltrazuril and gleptoferron (Forceris^®^ – CEVA) for the control of coccidiosis due to *Cystoisospora suis* was investigated in neonatal piglets. The study was carried out on five European commercial pig farms in France, Germany and Spain and 122 litters consisting of 1508 piglets were selected and randomly allocated to one of two treatment groups. The first group received a single intramuscular injection per piglet of the test product, containing 45 mg toltrazuril and 200 mg iron and this was compared with a control group, which was administered a single intramuscular treatment of iron at 200 mg per piglet only. Body weights, faecal scores and oocysts counts, recorded as oocysts per gram of faeces, (OPG) were observed for 21 days. Only 1138 piglets were actually exposed to coccidiosis and data sets of these animals were selected for statistical analysis. The efficacy of the test product in the control of coccidiosis was shown in higher body weight gains, a lower percentage of animals with diarrhoea, fewer samples with positive oocysts counts as well as a lower excretion peak and a reduced area under the curve of OPG from study day (SD) 4 – SD 21. The combination product of toltrazuril and gleptoferron provided an effective alternative approach to current conventional separate treatment for the prevention of iron deficiency anaemia and coccidiosis in neonatal piglets. It reduced the numbers of potentially stressful interventions and work time.

## Highlights

• Efficacy of Forceris^®^ against piglet coccidiosis successfully demonstrated.
• Treated piglets show reduced diarrhoea and oocysts excretion.
• Forceris^®^ treatment is safe and improves body weight gain.
• Combination of two active agents in one product reduces the numbers of stressful interventions and work time.

## 1 Introduction

Neonatal coccidiosis caused by *Cystoisospora suis* occurs in association with pig husbandry worldwide (Sayd and Kawazoe, 1996, Mundt et al., 2005a, Aliaga-Leyton et al., 2011, Zhang et al., 2012). Mundt et al. (2005b) published an overall farm prevalence of 69% in a large-scale study in 12 European countries. For instance, in Germany 83% of farms were tested positive by Niestrath et al. (2002). Clinical coccidiosis is characterised by yellowish rarely grey liquid, mostly pasty faeces, reduced body weight gains or weight loss and appears frequently at the age of 7–11 days (Lindsay et al., 2012). Although morbidity is high, mortality is usually low. Because coccidiosis is often associated with reduced weaning weights (Lindsay et al., 1985) significant economic losses for the farmers are likely (Scala et al., 2009, Kreiner et al., 2011).

Efficient disinfection (Straberg and Daugschies, 2007) and optimisation of management to avoid transmission of *C. suis* between litters and pens (Sotiraki et al., 2008) are important aspects in the control of piglet coccidiosis; however, even in pig production units with above-average hygiene outbreaks have been reported (Meyer et al., 1999).

In addition to the treatment against *C. suis*, several interventions related to farm management and health protection, e.g. vaccination against *Mycoplasma hyopneumoniae* (Maes et al., 2008) and routine castration are conducted during the first weeks of the piglets’ life. Supplementation of iron via intramuscular injection is also a common practice (Kegley et al., 2002, Szabo and Bilkei, 2002) in the phase of rapid neonatal growth, when iron deficiency will invariably lead to anaemia (Svoboda and Drabek, 2005). Since animal manipulation is needed for all these interventions, reduced handling for application of therapeutic compounds at the same time e.g. iron supplementation and anticoccidial therapy may reduce stress for the piglets. A number of studies have demonstrated the suitability of toltrazuril treatment to avoid disease and production losses due to coccidiosis. Toltrazuril treatment reduces not only the level and duration of oocyst excretion but also the risk of diarrhoea (Mundt et al., 2007, Joachim and Mundt, 2011) and may reduce the need for antibacterial treatments (Driesen et al., 1995). Furthermore, toltrazuril treated piglets display a better feed conversion (McOrist et al., 2010) and body weight development after weaning (Rypula et al., 2012). As a result, the farmer benefits economically as stated by Maes et al. (2007).

Recently a first injectable combination product, Forceris^®^ (30 mg toltrazuril/ml; 133.4 mg iron/ml as gleptoferron - CEVA) has been developed for the control of piglet coccidiosis and the prevention of iron deficiency anaemia. Treatment is scheduled from the first to the third day of life (24–96 h after birth) as a single intramuscular injection of a fixed dose of 1.5 ml/piglet corresponding to 45 mg of toltrazuril and 200 mg of iron.

The efficacy of Forceris^®^ was confirmed in comparison with an established reference product (oral toltrazuril suspension; Baycox^®^ 5% - Bayer Animal Health) by Joachim et al. (2018b). Results were obtained in an experimental infection model only, described by Mundt et al. (2006), but field data needed to be generated.

The aim of the multicentric study was to evaluate the efficacy of Forceris^®^ against *C. suis* in piglets under field conditions on farms with confirmed coccidiosis.

## 2 Material and methods

### 2.1 Trial design

The trial was designed as a multicentric, randomised, blinded study in compliance with the Guideline VICH GL9 relating to Good Clinical Practice. Two parallel groups were formed. Piglets treated by the test product once intramuscularly with 45 mg toltrazuril plus 200 mg iron per piglet at either day 1, 2, or 3 of age (IVP group). Piglets in the control group (CP) were treated by intramuscular injection of iron (200 mg/piglet) at day 1 of age only. The treatment was allocated per litter in order to be in accordance with real field conditions. The trial was conducted between October 2015 and February 2016. Five farms with a recent history of *C. suis* infection (confirmed oocyst excretion and/or pasty diarrhoea at an age of 7–21 days) were selected with the following designations: EC1 and HP1 in France, KB1 and KB2 in Germany and GC1 in Spain.

The main characteristics of the farms are listed in Table 1. Altogether 122 litters were included in the study and randomisation was applied at the day of farrowing. Only litters with more than 7 piglets were included. Of the 1688 piglets born alive, 1508 piglets were included, treated (761 piglets in the IVP group and 747 in the CP group) and followed according to the study protocol. Based on the farrowing management applied on the selected farms, treatment was administered on a fixed day in the week (at day 1, 2 or 3 of age, respectively). Distribution of male and female piglets between groups was similar (50.4% male piglets in IVP group and 49.3% in CP group). Treatment was not administered to piglets with signs of diarrhoea, impaired health or malformations. These animals were kept in the litter, but were treated with oral toltrazuril and 200 mg intramuscular injection of iron (gleptoferron). Over the study period piglets in EC1 (n = 264, 24 litters, 142 piglets in IVP group, 122 in CP group) and one batch of piglets on farm HP1 (n = 86, 7 litters, 53 piglets in IVP group, 33 in CP group) were found not to have been exposed to coccidiosis. These piglets were excluded from the statistical analysis for efficacy but were included for the safety evaluation. As a consequence, data from only 1138 piglets were incorporated for efficacy assessments. Piglets were monitored daily regarding health deteriorations over the whole study period and the injection site was inspected daily between study day (SD) 2 and SD 6 to assess the local tolerability of the product on the respective skin area (local tolerance). All observations were recorded and served to assess safety.

**Table 1:** Characteristics of the farms including farm type, breed, number of animals and litter size.

Farm ID	EC1	GC1	HP1	KB1	KB2	Total
Farm type	Farrow to Finish	Multi-site Farrowing unit	Farrow to Finish	Multi-site Farrowing unit	Farrow to Finish	
Breed	Female: Large white x Landrace Male: Pietrain	Large white x Landrace	Naima	Danish origin	Danish origin	
Number of included litters	24	30	18	24	26	122
Interval farrowing-weaning	19–23 days	21–24 days	18–23 days	23–27 days	23–27 days	
Number of piglets born per sow (mean ± SD)	16.3 ± 3.3	15.3 ± 2.5	17.4 ± 2.9	18.0 ± 3.0	17.7 ± 1.8	16.9 ± 2.9
Number of piglets born alive per sow (mean ± SD)	15.2 ± 3.0	14.4 ± 2.5	15.4 ± 2.9	14.3 ± 2.7	14.0 ± 1.0	14.6 ± 2.5
Number of included a (analyzed) b piglets	271	363	226	291	357	1508
	(0)	(346)	(127)	(264)	(344)	(1138)

a Data of included piglets (treated, followed according to the study protocol) were considered for safety evaluation.

b Number in parenthesis refer to those piglets that were incorporated for efficacy assessments.

**Fig. 1:**
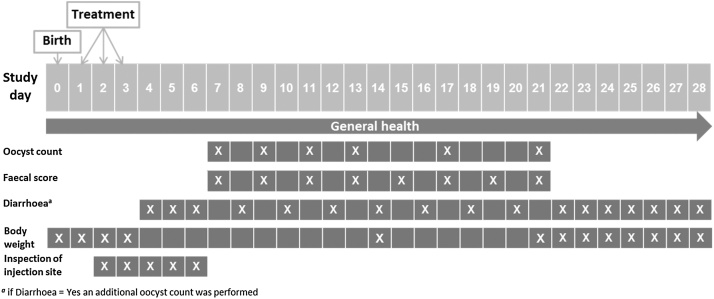
Study timeline and time points (ticked boxes) of study procedures during the period Study day (SD) 0 to SD 28.

A detailed study timeline is presented in Fig. 1. All piglets were checked individually for external signs of diarrhoea (e.g. smeared anus) on the SDs (between SD 4 and the end of the study), when faecal sampling was not scheduled per protocol. In case diarrhoea was suspected, an additional faecal sample was taken and an oocyst count was performed.

### 2.2 Efficacy parameters and statistics

The protocol of this study was in accordance with the recently published guideline on evaluation of efficacy of anticoccidials in piglets and other mammalian species (Joachim et al., 2018a). Statistical calculations were performed using SAS® version 9.3 (SAS Institute Inc., Cary, USA) for all included, randomised and treated piglets, that were exposed to coccidiosis. A quantitative determination of oocyst numbers (oocysts per gram faeces, OPG) was performed for each piglet six times during the study (Fig. 1) by using a modified McMaster counting method (Mundt et al., 2006). At each sampling date, descriptive statistics of the OPG counts (including arithmetic and geometric mean, median and number of samples with positive OPG counts) were applied for each group. In addition, the area under the curve (AUC) was calculated for the OPG counts over the period SD 4 to SD 21 and was compared between groups by analysis of variance with group and farm as fixed effects. During the study period consistency of faeces was recorded for each piglet eight times (Fig. 1) according to the following scores: 1 = firm, 2 = pasty, 3 = semi-liquid, 4 = liquid. Diarrhoea was defined as a faecal score (FS) > 2. For both OPG and FS the percentage of piglets was compared between groups by bilateral testing. A generalized linear model for binomial counts was used, taking into account the expected correlation between observations on piglets in the same litter.

Success of treatment was defined as follows: 1) Piglets without oocyst shedding and with at least three samples available for McMaster counting or 2) piglets that never displayed diarrhoea and with at least four FS values recorded.

Body weight (BW) was measured five times for each piglet (Fig. 1). BWs on SD 0 were compared between groups using student’s t - test. The mean daily body weight gain (MDBWG) was calculated for the periods from initial weighing until SD 14 and SD 21. Values were compared between groups by covariance (ANCOVA) analysis.

Mortality rates were compared between groups using chi - square - test.

P - values ≤ 0.05 were considered as statistically significant.

## 3 Results

### 3.1 Safety

Three out of 761 piglets (0.4%) of the IVP group showed transient local reactions at the injection site. One animal displayed shock symptoms and another became lethargic after treatment. All piglets recovered without any veterinary interventions. Total pre-weaning mortality was 9.1%, whereas a significantly lower (p = 0.046) number of piglets died in IVP group (7.6%) compared to group CP (10.6%).

### 3.2 Body weight (BW), mean daily body weight gain (MDBWG)

BWs on SD 0 were similar between groups (IVP group: mean 1431.7 ± 342.1 g; group CP: mean 1412.9 ± 329.3 g) without any significant difference (p = 0.359). Therefore the parameter MDBWG could be used for evaluation of efficacy. On SD 14 and SD 21 BWs were higher in the IVP group (4279.6 ± 1272.3 g and 5936.9 ± 1821.1 g) compared to group CP (4115.5 ± 1153.3 g, 5625.6 ± 1660.3 g). The same applied to MDBWG with significant higher values in the IVP group for the period SD 0 to SD 14 (p = 0.034) and SD 0 to SD 21 (p = 0.004) as presented in Table 2.

**Table 2:** Mean daily body weight gain (MDBWG) of piglets treated by a combination of toltrazuril and iron (IVP group) in comparison to piglets treated by iron (CP group).

	IVP group	CP group	p-value
MDBWG SD^a^ 0 – SD^a^ 14			
Mean ± SD	202.8 ± 74.3	192.6 ± 71.1	0.034
[Min, Max]	[16.9, 435.0]	[30.0, 392.9]	

MDBWG SD^a^ 0 – SD^a^ 21			
Mean ± SD	213.7 ± 77.3	200.1 ± 72.9	0.004
[Min, Max]	[34.8, 453.3]	[21.4, 404.8]	

a Study day (SD).

### 3.3 Oocyst excretion

The efficacy of the test product to control oocyst excretion was proven for all farms were coccidiosis was observed. The probability for piglets not to excrete oocysts was distinctly increased in the IVP group (lower bound of 95% confidence limit of Odds Ratio > 1).

**Fig. 2:**
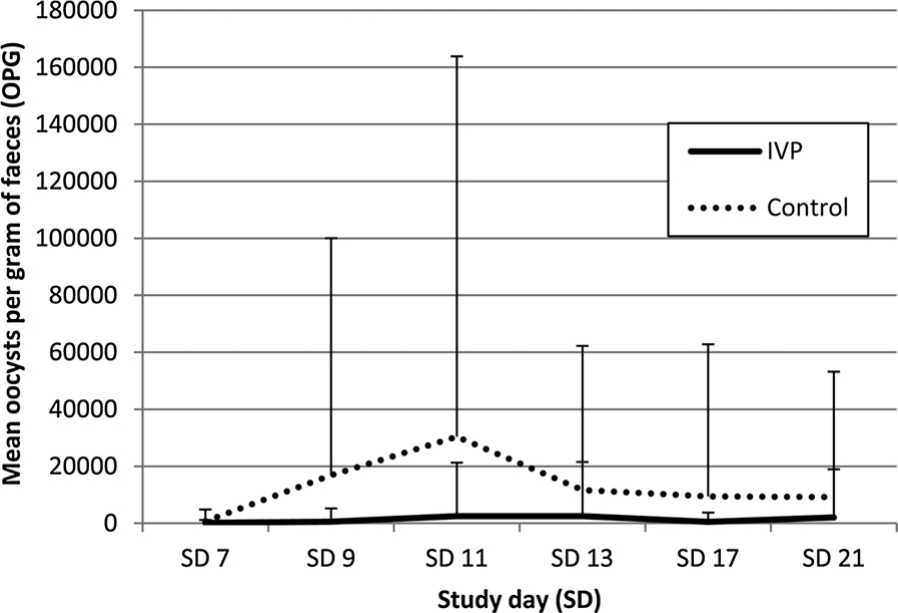
Mean oocyst excretion in OPG (oocysts per gram of faeces) on Study day (SD) 7, SD 9, SD 11, SD 13, SD 17 and SD 21 for piglets treated by a combination of toltrazuril and iron (IVP) and piglets treated by iron (Control).

The course of oocyst excretion by treatment group is shown in Fig. 2. Oocyst counts were consistently higher in the CP group with a peak mean value of 30403.7 OPG at SD 11. In the IVP group peak of mean oocyst excretion was reached two days later on SD 13 and was distinctly lower with 2506.2 OPG.

**Fig. 3:**
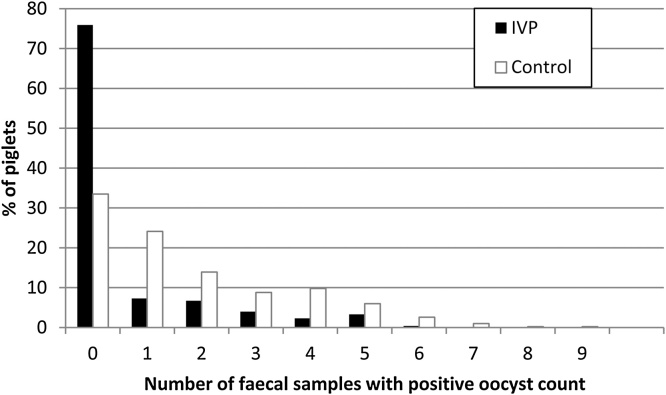
Number of faecal samples with positive oocyst count by treatment group.

In the IVP group the proportion of piglets that never excreted oocysts (75.9%) during the investigation period was significantly higher (p < 0.001) compared to group CP (33.5%), although some variation between farms was observed (Table 3). The number of faecal samples with positive oocyst count was reduced in piglets treated with the test product (0.6 samples on average with a maximum of 6 samples from two piglets) in comparison to group CP (1.7 samples on average with a maximum of 9 samples from one piglet) (Fig. 3).

**Table 3:** Percentage of piglets treated by a combination of toltrazuril and iron (IVP) in comparison to piglets treated by iron (CP) with positive oocyst counts and area under the curve (AUC) for the period Study day (SD) 4 to SD 21 scaled per farm.

	Farm HP1		Farm GC1		Farm KB1		Farm KB2		Overall	
	IVP	CP	IVP	CP	IVP	CP	IVP	CP	IVP	CP
Percentage of piglets with oocyst excretion (number of piglets/total)	0.0 % (0/45)	60.3 % (35/58)	67.5 % (110/163)	97.0 % (160/165)	1.0 % (1/105)	51.7 % (61/118)	2.4 % (4/165)	48.4 % (78/161)	24.1 % (115/478)	66.5 % (334/502)
AUC (SD 4 - SD 21) Mean ± SD	0.0 ± 0.0	15.6 ± 19.6	29.9 ± 30.3	69.1 ± 34.8	0.3 ± 2.6	18.9 ± 24.8	0.4 ± 2.8	20.4 ± 28.2	10.4 ± 22.6	35.2 ± 37.2

The AUC of oocyst excretion was significantly lower in the IVP group in comparison with group CP (p < 0.001; Table 3).

### 3.4 Diarrhoea

**Fig. 4:**
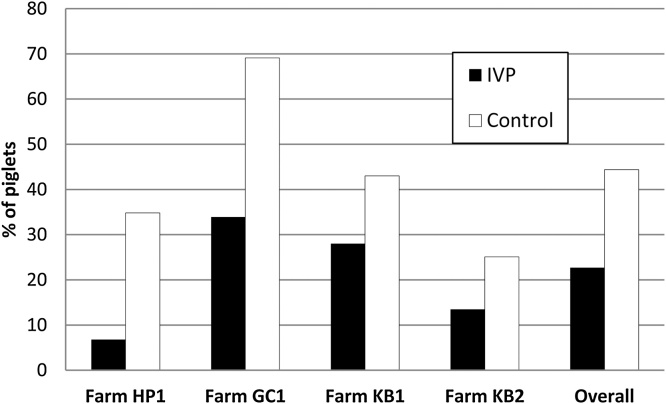
Percentage of piglets presenting diarrhoea at least once in individual farms by treatment group.

The success of treatment with the test product regarding the control of diarrhoea was clearly demonstrated (22.7% of the piglets presenting diarrhoea at least once in the IVP group versus 44.4% in group CP, p < 0.001; Fig. 4).

## 4 Discussion

In a controlled, randomised, blinded study, the efficacy of the test product to control coccidiosis in suckling piglets exposed to natural infection with *C. suis* was assessed. The study was conducted in three major pig producing countries in the European Union: Spain, Germany and France (Eurostat, 2017), including farms with different management and organisation of production and recently confirmed history of coccidiosis. In all chosen countries coccidiosis is considered as a frequent and major cause of suckling piglet diarrhoea (Torres, 2004, Mundt et al., 2005a, Mundt et al., 2005b, Ruiz et al., 2016). In total, the study population consisted of 122 litters with 1508 piglets fulfilling the inclusion criteria, and thus we believe that the data are representative for conventional piglet rearing facilities in Europe.

*C. suis* infection often results in reduced body weight gains in young piglets (Lindsay et al., 1985). Rypula et al. (2012) and Mundt et al. (2003b) demonstrated the positive effect of toltrazuril treatment on body weight gains in infected piglets. In agreement with a previous report on Forceris® treatment of experimentally infected piglets (Joachim et al., 2018b), we confirmed significantly improved body weight gains in naturally coccidiosis exposed piglets.

Toltrazuril is well known to be highly effective in the control of piglet coccidiosis, however, in our study the route and time point of administration differed from the registered oral application (3–5 days of life). The success of oral treatment was already demonstrated for toltrazuril by various studies (Driesen et al., 1995, Skampardonis et al., 2010, Kreiner et al., 2011, Rypula et al., 2012). Our parasitological data clearly illustrate that a single application of the test product within the first three days after birth efficiently reduced OPG and the number of faecal samples with positive oocyst counts. Piglets may become infected during the first days after birth with the consequence of intestinal lesions developing coincidentally with the onset of oocyst excretion (Mundt et al., 2006). Treatment at day 1, 2, or 3 after birth will disrupt the parasite’s life cycle early enough to prevent these lesions. Moreover, immediate disruption of parasite reproduction prevents environmental contamination with oocysts and thus reduces infection pressure (Mundt et al., 2003a, Mundt et al., 2003b).

As expected, the proportion of piglets suffering from diarrhoea was lower when the test product was applied. This beneficial effect confirms previous studies where toltrazuril was administered per os to experimentally infected (Mundt et al., 2003b, Mundt et al., 2007, Joachim and Mundt, 2011, Joachim et al., 2018b) or naturally exposed piglets (Skampardonis et al., 2010, Kreiner et al., 2011, Streyl et al., 2015). It is generally recognised that *C. suis* is an important enteropathogen in piglets. However, under field conditions other enteropathogens e.g. *Escherichia coli* (Ruiz et al., 2016) or *Clostridium perfringens* (Vítovec et al., 1991, Ruiz et al., 2016) may also cause diarrhoea in suckling piglets. In fact, 22.7% of the piglets that were treated with the test product still displayed diarrhoea. Unfortunately, differential diagnostics including evaluation of virulence factors were not performed and therefore the cause of diarrhoea, which was not due to *C. suis* as no oocyst excretion could be observed, remains unclear.

In this multicenter study *C. suis* infection rates varied considerably between farms (38.5% (HP1) to 97.0% (GC1) of untreated controls). Such variations were reported earlier and obviously correspond to farm management and hygiene measures (Aliaga-Leyton et al., 2011, Skampardonis et al., 2012). Regional aspects may also have an impact on coccidiosis prevalence (Meyer et al., 1999). Efficacy of the test product was demonstrated irrespective of the different conditions and infection pressure on the selected farms. Similar to Kreiner et al. (2011) oocyst excretion was completely prevented (HP1) or was very low (KB1: 1.0%; KB2: 2.4%) in toltrazuril treated piglets under field conditions. Most strikingly, 67.5% of treated piglets on farm GC1 excreted oocysts at least once during the observation period. This may reflect an extreme level of infection pressure on this particular farm, as indicated by the very high prevalence of *C. suis* (97%) in group CP. Not only high infection pressure, but also a low susceptibility to toltrazuril of the *C. suis* strain circulating on farm GC1 might explain that two-thirds of piglets treated with the IVP shed oocysts. Shrestha et al. (2017) confirmed resistance of a *C. suis* field strain in experimentally infected piglets treated with toltrazuril. Concerning oocysts excretion, faecal consistency and body weight gain a distinct lack of efficacy of toltrazuril was observed by these authors. Contrary to Shrestha et al. (2017) in the current study the efficacy of the test product was demonstrated and oocyst excretion was considerably reduced on all farms and even in a highly contaminated environment.

Mortality data generated in the current study are similar to those published by Thanner and Gutzwiller (2018) and Welp (2014), but can vary considerably up to 20% and more (Mainau et al., 2015, Kielland et al., 2018, Nuntapaitoon et al., 2018). The group comparison revealed a significant lower mortality in the IVP group than in group CP. Moreover, a local injection site reaction after application of the test product occurred in only 0.4% of the piglets. Therefore, the safety and good local tolerance of the intramuscular injection of the combination of toltrazuril and iron in one product, Forceris^®^, is confirmed by this extensive trial data.

Albeit the comparison between intramuscular injection of the test product and oral administration of toltrazuril was not the focus of this study, it can be assumed that intramuscular application allows more precise and reliable dosing, especially if piglets are vomiting or do not tolerate oral application. Toltrazuril treatment early after birth has been suggested to prevent development of necrotic enteritis due to associated infection by clostridia in affected herds (Mengel et al., 2012) and the test product might be suitable for this particular indication, however, this remains to be investigated.

## 5 Conclusion

The results of this multicentric, randomised, blinded study in piglets naturally infected with *C. suis* clearly demonstrated the efficacy of Forceris^®^ at a fixed dose of 1.5 ml/piglet (45 mg toltrazuril plus 200 mg iron) when administered once at either day 1, 2, or 3 of age. Coccidiosis due to *C. suis* was successfully controlled as Forceris^®^ treated piglets displayed significantly reduced oocyst excretion, less diarrhoea and improved body weight gain. The injectable combination of toltrazuril and iron administered early after birth is a safe and efficient option in order to control coccidiosis and to supplement iron at the same time. This reduces work load and stress exposure of piglets due to repeated handling on farms where piglet coccidiosis is a relevant problem.

## Funding

This study was funded by CEVA, France.

## Conflict of interest

The authors declare that they have no conflict of interest. Daniel Sperling, Gaëlle Pagny, Laurianne Meppiel and Naomi Isaka are employees of CEVA. No member of the staff of Albrecht - Daniel - Thaer - Institute received any personal benefits from the Sponsor.

All procedures were approved by the institutional ethics committees and the national authorities according to respective national laws (reference/approval numbers: Germany V 244 - 75085/2015; France ENR/KLD EC-00776-0; Spain 364/ECV).

## Data statement

Raw data will not be shared as study documentation is protected by confidentiality agreements.
